# The association of 9p21-3 locus with coronary atherosclerosis: a systematic review and meta-analysis

**DOI:** 10.1186/1471-2350-15-66

**Published:** 2014-06-06

**Authors:** Muhammad S Munir, Zhen Wang, Fares Alahdab, Mark W Steffen, Patricia J Erwin, Iftikhar J Kullo, Mohammad Hassan Murad

**Affiliations:** 1 Division of Preventive Medicine, Mayo Clinic, Rochester, MN, USA; 2 Hospital Medicine, University of Wisconsin Medical Foundation, Madison, WI, USA; 3 Knowledge and Evaluation Research Unit, Mayo Clinic, Rochester, MN, USA; 4 Mayo Clinic Libraries, Mayo Clinic, Rochester, MN, USA; 5 Division of Cardiovascular Medicine, Mayo Clinic, Rochester, MN, USA

**Keywords:** Coronary, Atherosclerosis, 9p21-3

## Abstract

**Background:**

Studies suggest that the 9p21-3 locus may influence susceptibility to myocardial infarction. We performed a systematic review and meta-analysis to assess whether this locus is associated with severity of coronary atherosclerosis and adverse clinical outcomes in those with known coronary disease.

**Methods:**

Multiple electronic databases were searched from inception through August 2012. Studies examining 9p21-3 genotype in patients with known coronary artery disease were included. We extracted the association of the 9p21-3 locus with measures of severity of coronary atherosclerosis [number of diseased vessels, Gensini Score, Duke CAD Prognostic Index (DPI)], angiographic outcomes [change in minimum lumen diameter (∆MLD) and number of new lesions at follow-up], and key clinical outcomes (all-cause mortality, recurrent myocardial infarction and the need for coronary revascularization). Relative risks (RR) and weighted mean difference (WMD) were pooled using the random effects models.

**Results:**

23 cohorts enrolling 16,860 participants were analyzed. There was no significant difference between HR and LR genotypes in terms of all-cause mortality, recurrent myocardial infarction or the frequency of coronary revascularization. HR genotype was associated with increased risk of triple vessel disease (RR = 1.34; 95% CI 1.08-1.65; P = 0.01) and increased baseline Gensini Score (WMD = 5.30; 95% CI 0.66-9.93; P = 0.03). However there was no association with DPI (WMD = 4.00; 95% CI 2.94-10.94; P = 0.26). HR genotype did not predict ∆MLD or number of new lesions at follow-up.

**Conclusions:**

Patients of coronary atherosclerosis who carry the high risk genotype of the 9p21-3 allele may be more likely to have multi-vessel CAD. However the effect of this allele on CAD progression and disease specific clinical outcomes are not observed possibly due to diminishing genetic risk following dietary modification and therapy.

## Background

Coronary artery disease (CAD) remains a worldwide leading cause of mortality. Modification of major environmental risks such as smoking and high cholesterol reduces CAD mortality by 20% to 30% [1]. The presence of a positive family history as a strong risk factor in CAD points to underlying genetic risk factors [2].

Genome wide association studies (GWAS) have identified over 30 risk variants for CAD [3,4]. Of these, the variant on the p arm of chromosome 9 at position 21–3 (9p21-3) is the most well-known and replicated. Many studies have established and replicated the association of the 9p21-3 locus with CAD and myocardial infarction (MI). Other studies have revealed that targeted deletion of the 9p21 non-coding interval leads to excessive proliferation of vascular smooth muscle cells as well as their diminished senescence [5]. Some 9p21 variants also impair the inflammatory response in vascular cell types, which might explain some of the genetic susceptibility underpinning CAD [6]. Variants at this locus have also been associated with a lower ankle-brachial index (ABI), which is a marker of increased risk for death and incident cardiovascular disease (CVD) events [7]. The effect of the 9p21-3 locus on angiographic severity and clinical outcomes in patients with established CAD has been tested by several investigators. However, findings from these reports are conflicting.

We therefore conducted a systematic review and meta-analysis of the published literature investigating the association of the 9p21-3 locus with angiographic CAD severity, progression, and key clinical outcomes.

## Methods

The reporting of this systematic review complies with the Preferred Reporting Items for Systematic Reviews and Meta-Analyses (PRISMA) statement [8].

Eligible studies were comparative studies of human subjects, provided genotyping was done at the 9p21-3 locus in a population with known coronary artery disease (previous/recent MI, or known epicardial coronary stenosis at enrollment). Applicable study designs included observational studies (case–control, cohort and cross sectional) where an association between the 9p21-3 allele and poor outcome or prognostic marker was reported. Only studies written in English were included due to feasibility.

We searched Ovid MEDLINE from 1948 until August 2012 and Ovid EMBASE, Web of Science and SCOPUS, from inception to August 2012. Subject headings (MeSH, EMTREE) were used: Chromosomes, Pair 9, Coronary artery disease, alleles and atherosclerosis. Keywords (9p21*) were used in Web of Science and Scopus. The detailed search strategy is attached in Additional file 1.

A team of two trained reviewers independently screened all articles identified in the literature search. Discrepancies between the reviewers were resolved through discussions and consensus.

Markers of atherosclerotic severity included number of diseased vessels, Gensini Score and Duke CAD Prognostic Index (DPI). Markers of atherosclerotic severity and coronary disease progression are defined elsewhere [9]. We also assessed change in minimum lumen diameter (∆MLD) and number of new lesions at follow-up. Outcomes of interest included all-cause mortality, recurrent MI, need for coronary revascularization, triple vessel disease, Gensini score, DPI, ∆MLD, and number of new lesions. In studies where all-cause and cause-specific mortalities were separately tested, we analyzed all-cause mortality only.

Recurrent MI was defined any acute coronary syndrome associated with troponin elevation and/or ST segment elevation on electrocardiography (ECG). Need for coronary re-vascularization included surgical and percutaneous procedures performed either at target or non-target coronary vessels.

We extracted details on sample size, mean age, race, the identification (rs number) of the particular SNP genotyped, and outcomes of interest. SNPs previously reported in GWAS studies or in strong linkage disequilibrium with them were considered in the analysis.

In keeping with our goal to determine locus-outcome association we did not limit our analysis to a single SNP but instead tested for all available SNPs published in reports chosen for the meta-analysis. In studies reporting > 1 SNP-outcome association, we chose the SNP not elsewhere tested in other data sets. This allows us to capture all known markers in the locus and test as many markers as possible.

We used the Newcastle-Ottawa Quality Assessment to assess the risk of bias of the included studies [10]. The following items were used: selection of patients, comparability, assessment of exposure and/or outcome, length of follow-up, lost to follow-up. We were unable to assess potential publication bias due to limited number of studies included for each outcome [11].

Genotypes were classified as either homozygous low risk (LR) heterozygous intermediate risk (IR) or homozygous high risk (HR). Study results were variedly reported using recessive [LR vs. (IR + HR)], dominant [(LR + IR) vs. HR)] and additive models [LR vs. IR vs. HR]. For the purpose of this manuscript we included additive models. For dichotomized outcomes, we extracted or calculated relative risk (RR) and its 95% confidence intervals (CI). We then pooled RR across the studies using the DerSimonian and Laird random effects methods with the heterogeneity from the Mantel–Haenszel method [12]. For continuous outcomes, we pooled weighted mean difference (WMD) using the same DerSimonian and Laird random effects methods.

We assessed the optimal information size (OIS), similar to power calculation in clinical trials, to evaluate the minimum sample size required in the literature to reach reliable conclusions [8].

We assessed the consistency of the outcomes by testing heterogeneity using the *I*^*2*^ statistic, where *I*^*2*^ > 50% suggests a high level of heterogeneity [13]. All statistical analyses were conducted using STATA version 12 (StataCorp, College Station, TX).

## Results

**Figure 1:**
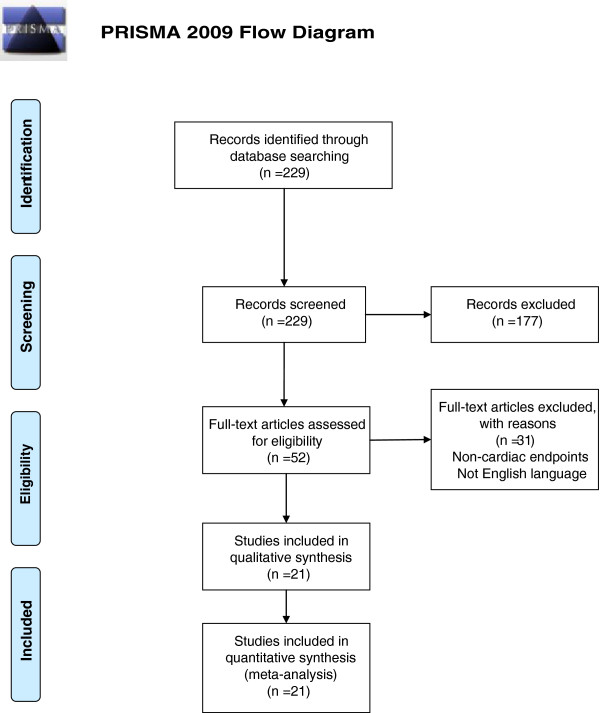
Flow Chart: PRISMA 2009 Flow Diagram.

The literature search yielded 229 studies of which 21 (describing 23 distinct cohorts) met criteria for inclusion. Study selection process is described in (Figure 1). Table 1 lists the studies entered in the meta-analysis together with outcomes tested in each study.

**Table 1:** Characteristics of the included studies

**Study ID**	**Genotyping method**	**SNP: and base position**	**Minor allele frequency**	**Linkage disequilibrium (LD) (reported as D’ or r**^**2**^**)**	**Population (age/(%)male/ethnicity)**	**Baseline diagnosis**	**Outcome/prognostic marker (1)**	**Outcome/prognostic marker (2)**	**Outcome/prognostic marker (3)**	**Comments**
Anderson, [14]	with 5′ exonuclease (Taqman) chemistry on the ABI Prism 7000	rs2383206	0.48		1759 (59/64%/mixed race with 86% white)	CAD	No. diseased vessels	baseline Duke index		Prospective cohort
Chen, [15]	fluorogenic 5′ nucleotidase (Taqman) assays using an ABI PRISM® 7900 HT Real-Time PCR instrument	rs2383206 (chromosomal position: 22,105,026), rs2383207 (chromosomal position: 22,105,959), rs10757278 (chromosomal position: 22,114,477), rs1333049 (chromosomal position: 22,115,503)	rs2383206 = 0.15	D’ rs2383206 – 2383207 = 0.99	212 (48/58%/Chinese)	CAD	No. diseased vessels			Case control
				D’rs2383206-10757278 = 1.0						
				D’rs2383206 – rs1333049 = 0.99						
Chen, [16]	TaqMan allelic discrimination assays	rs7865618: 22021005	0.38	LD of rs7865618, rs1537378, rs1333040 with rs1333049 0.81 ≤ r^2^ ≤ 0.97	322 (59/84%/white)	CAD	∆MLD	no. of new lesions		Case control
Hoppman, [17]	TaqMan allelic discrimination assays	rs7865618: 22021005	0.38	LD of rs7865618, rs1537378, rs1333040 with rs1333049 0.81 ≤ r^2^ ≤ 0.97	2028 (−−/−−%/white)	CAD/ACS	All-cause mortality	Recurrent MI	revascularization	Prospective cohort
		rs1537378: 22051614	0.35							
		rs1333040: 22073404	0.40							
		rs1333049: 22115503	0.49							
Peng, [18]	TaqMan single nucleotide polymorphism	rs1333049	0.24	rs10757274 and rs1333049 had strong LD (r^2^ = 0.92)	520 (64/78.7%/Chinese)	ACS	All-cause mortality	Recurrent MI	No. diseased vessels	Case control
Newton-Cheh, [19]	Sequenom platform (San Diego, Calif), which resolves allele-specific single-base extension products using mass spectrometry (MALDI-TOF)	rs10757274 backup: rs2383207	Case: 0.54 Control: 0.50	Rs10757274 and rs2383207 are in strong linkage disequilibrium (*r*^2^ = 0.87).	466 (−−/--%/whites)	CAD	SCD			Retrospective case–control study of SCD†. Subgroup analysis compared 124 SCD cases with 342 controls. Both cases and controls had CAD
Ellis, (1) [20] (CDCS)	allelespecific TaqMan genotyping probes	rs1333049	GG: 0.22		860 (67/69.2%/whites)	ACS	All-cause mortality	Recurrent MI		Cohort study
Ellis, (2) [20] (PMI)	allelespecific TaqMan genotyping probes	rs1333049	GG: 0.22		607 (62/78.6%/whites)	ACS	All-cause mortality	Recurrent MI		Cohort Study
Buysschaert, [21]	iPLEX technology on a MassARRAY	rs1333049	0.25		2942 (65/67.9%/whites)	ACS	Recurrent MI			Prospective cohort
		rs7044859								
		rs1292136								
		rs7865618								
	Compact Analyser (Sequenom Inc., CA, USA). The WTCCC controls: Affymetrix platform (Affymetrix Inc., CA, USA).									
Patel, [22]	Centaurus (Nanogen) platform	rs10757278	0.24		2334 (63/67%/whites)	CAD/ACS	No. diseased vessels	baseline Gensini score		Prospective cohort
Muehlschlegel, [23]	Golden Gate assay with an Illumina Bead Station 500G system (Illumina)	rs10116277	0.18		846 (−−/--%/whites)	CAD	All-cause mortality			Prospective cohort
Dandona, [24]	Affymetrix (Santa Clara, California) 500 K and 6.0 arrays	rs1333049	0.19	rs9632884 was linkage disequilibrium with rs1333049 (r^2^ = 0.832).	1714 (−−/--%/whites)	CAD/ACS	No. diseased vessels	baseline Duke index	baseline Gensini Score	case control
Liu, [25]	Golden Gate assay with an Illumina Bead Station 500 G system (Illumina, San Diego, Calif)	rs10116277			846 (−−/--%/whites)	CAD	Recurrent MI			Prospective cohort
		rs6475606								
		rs2383207: chromosom								
		e 9								
		positions 21,930,588								
		22,366,970								
Wang, [26]	TaqMan SNP allelic discrimination by means of an ABI 7900HT (Applied Biosystems, Foster City, CA, USA)	rs1333049			430 (−−/--%/Chinese)	CAD	∆MLD	no. new lesions		case control
Ardissino, [27]	Matrixassisted laser desorption ionization time-of-flight mass spectrometry and a Sequenome MassARRAY platform (San Diego, California)	rs1333040			1508 (41/95%/Italian)	ACS	All-cause mortality	recurrent MI	revascularization	Prospective cohort
Wang, [28]	TaqMan SNP allelic discrimination by means of an ABI 7900HT (Applied Biosystems, Foster City, CA, USA)	rs1333049: 9p21.3	0.25		620 (67/51.2%/Chinese)	CAD	No. diseased vessels	baseline Gensini score		Cross sectional
Chan, [29]		rs1333049			332 (59/84%/whites)	CAD	∆MLD	no. new lesions		Prospective cohort
Dutta, [30]	Conventional Taqman PCR (probes and assays designed by Applied Biosystems; Foster City, CA)	rs1333049			478 (75/--%/whites)	CAD	All-cause mortality			Propective cohort
Kozieradzka, [31]	TaqMan SNP Genotyping Assay using the ABI 7500 Real Time PC R System (Applied Biosystems)	rs1333049			582 (62/75%/--)	CAD	All-cause mortality			Cohort Study
		rs4977574								
		rs10757278								
Gioli-Pereira, [32]	Submicroliter PCR-based assay on array tape that is a continuous plastic tape used in conjunction with a flexible configuration of dispensing, pipetting, sealing and detection modules manufactured by Douglas Global Array	rs10757274, rs2383206, rs10757278, rs1333049	rs10757274: 0.51,		611 (60/84.9%/Brazilian)	CAD	All-cause mortality	No. diseased vessels		Prospective cohort
			rs2383206: 0.59,							
			rs10757278: 0.51,							
			rs1333049: 0.48							
Virani, (1) [33]	TaqMan assays	rs1333049,	CC: 0.27,	all 4 SNPs from our analyses (rs1333049, rs2383206, rs10757278, and rs10757274) have been shown to be in strong linkage disequilibrium (LD),	2067 (63/74%/whites)	ACS	All-cause mortality	recurrent MI	revascularization	Prospective cohort
			CG: 0.50,							
		rs2383206,								
			GG: 0.23;							
			CABG group:							
		rs10757278, rs10757274								
Virani, (2) [33]	TaqMan assays	rs1333049,	CC: 0.30,	all 4 SNPs from our analyses (rs1333049, rs2383206, rs10757278, and rs10757274) have been shown to be in strong linkage disequilibrium (LD),	1176 (65/79%/whites)	CAD	All-cause mortality	recurrent MI	revascularization	Prospective cohort
		rs2383206,								
		rs10757278, rs10757274	CG: 0.50,							
			GG: 0.21;							
Lill, [34]		rs2383206			452 (58/78%/--)	CAD	All-cause mortality			Cohort Study

CAD: coronary artery disease; ACS: acute coronary syndrome; SCD: sickle-cell disease; MI: myocardial Infarction.

**Figure 2:**
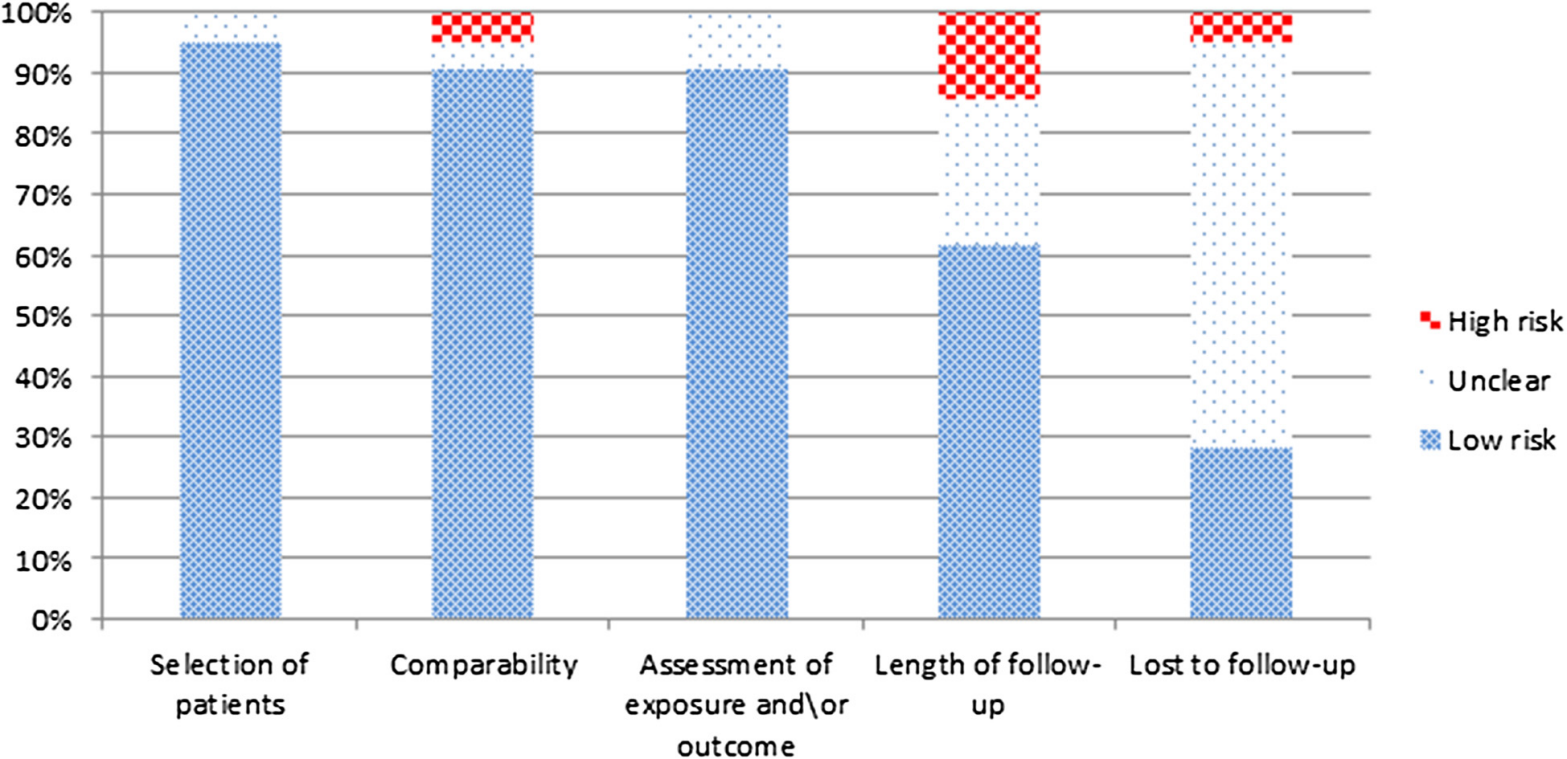
Risk of bias of the included studies.

The methodological quality of the included studies was fair, with the majority of them providing adequate representativeness of study patients, comparability between patient groups and sufficiently assessment of exposure and/or outcome (Figure 2). Also, in all of the outcomes, except all-cause mortality, triple vessel disease, and Gensini Score, the total sample size reported in the studies were less than the OIS. We, thus, were unable to reach conclusive findings for these outcomes.

**Figure 3:**
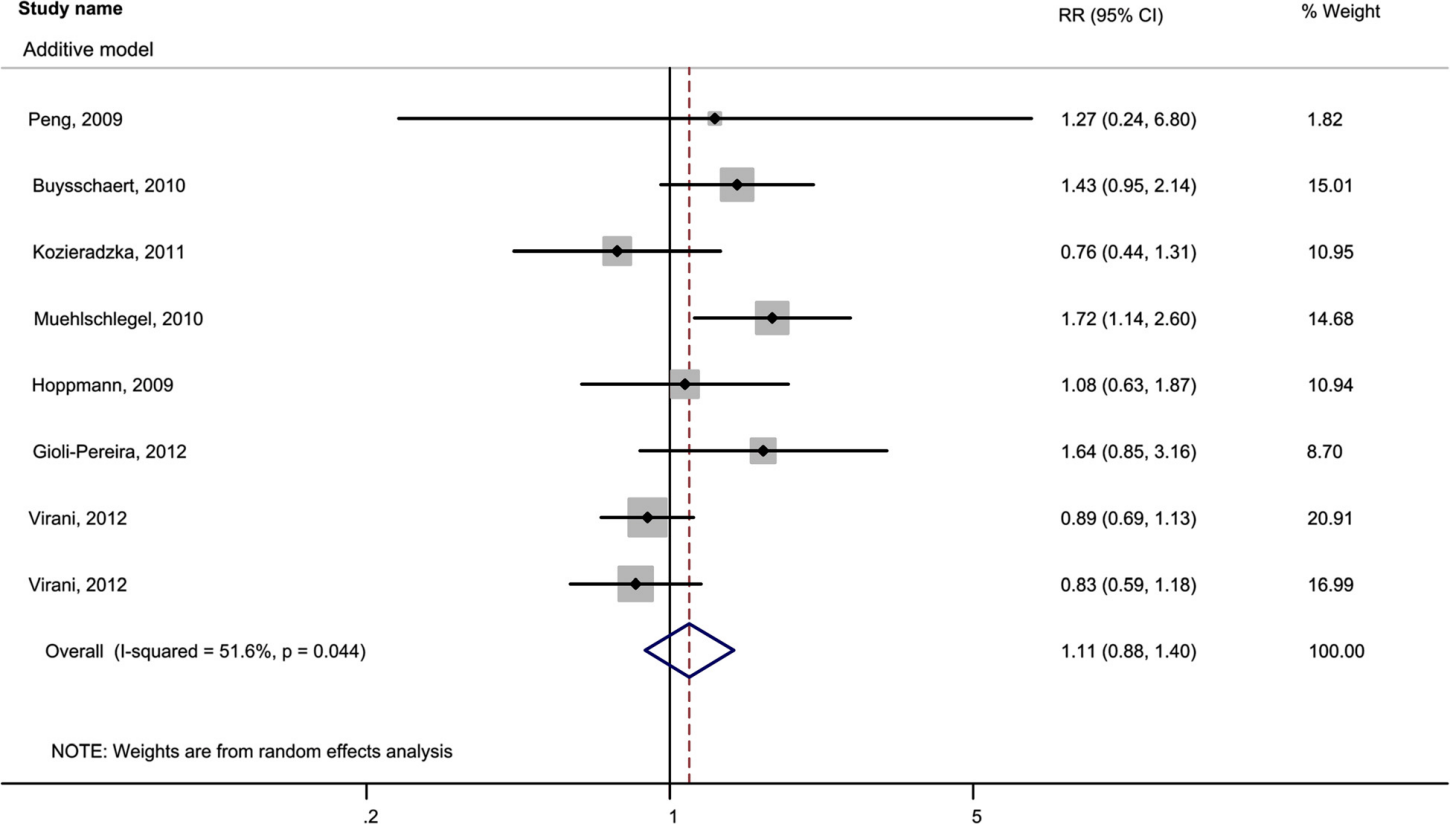
Pooled relative risk of all cause mortality using additive [LR vs. IR vs. HR], dominant [(LR + IR) vs. HR)], and recessive [LR vs. (IR + HR)] models.

We did not find a significant association between 9p21-3 and all-cause mortality (RR = 1.11; 95% CI 0.88-1.40; p = 0.39, I^2^ = 51.6%) (Figure 3).

Likewise, no significant association emerged in the meta-analysis of 9p21-3 with recurrent MI in patients with known CAD in the additive model (RR = 1.14; 95% CI 0.92-1.40; p = 0.24; I^2^ = 7.0%). Table 2 lists the summary statistics for the outcomes.

**Table 2:** Pooled statistics using additive [LR vs. IR vs. HR], dominant [(LR + IR) vs. HR)], and recessive [LR vs. (IR + HR)] models

	**Number of cohorts**	**Number of patients**	**RR**	**95% CI**		**P value**	**I**^**2**^
**Recurrent MI**	6	5795	1.14	0.92	1.40	0.24	7.0%
**Revascularization**	4	5225	1.11	0.78	1.57	0.56	78.1%
**Triple vessel disease**	4	1928	1.34	1.08	1.65	0.01	53.8%
			**WMD**	**95% CI**		**p.value**	**I**^**2**^
**Gensini Score**	3	1470	5.30	0.66	9.93	0.03	80.2%
**DPI**	2	1201	4.00	−2.94	10.94	0.26	87.5%
Δ**MLD**	2	293	0.07	−0.02	0.15	0.15	1.0%
**No. new lesions**	2	293	0.03	−0.05	0.10	0.49	0.0%

MI indicates myocardial infarction; DPI, Duke CAD prognostic index; ∆MLD, change in minimum lumen diameter; No. new lesions, number of new lesions.

Four cohorts from 3 studies reported need for re-vascularization. No significant association was identified between 9p21-3 and re-vascularization after development of CAD (RR = 1.11; 95% CI 0.78-1.57; p = 0.56; I^2^ = 78.1%).

The meta-analysis supported an association between 9p21-3 and triple vessel disease. Homozygotes (HR) for the risk allele had significantly greater risk (RR = 1.34, 95% CI 1.08-1.65, p = 0.01, I^2^ = 53.8%).

Three studies reported severity of CAD as measured by Gensini score at baseline. Combined analysis of these studies showed 5.30 higher mean Gensini score in the LR group vs. the HR group. This difference was significant (95% CI 0.66-9.93; p = 0.03; I^2^ = 80.2%). However the DPI which also quantifies CAD severity was not significant in the combined analysis of the two studies reporting it (WMD = 4.00; 95% CI −2.94-10.94; p = 0.26; I^2^ = 87.5%).

Combined analysis of two studies testing for association of angiographic progression as measured by Δ MLD and number of new lesions at follow-up revealed no association with the 9p21-3 allele. The combined WMD for Δ MLD was 0.07 (95% CI −0.02-0.15; p = 0.15; I^2^ = 1.0%) and new lesions at follow up was 0.03 (95% CI −0.05-0.10; p = 0.49; I^2^ = 0.0%).

## Discussion

In this meta-analysis of studies investigating angiographic severity and clinical outcomes in patients with CAD, we found an association of 9p21-3 allele with increased risk of triple vessel disease and greater quantitative severity of atherosclerosis as measured with the Gensini score at baseline. The meta-analysis did not support an association of the allele with angiographic outcomes at follow up or clinical outcomes.

Our findings are consistent with the results of Chan et al. [35] who reported a 23% greater risk of triple vessel disease among high risk homozygotes when compared with their low risk genetic counterparts. Different from Chan’s study, we analyzed more outcomes, including measures of severity of coronary atherosclerosis [number of diseased vessels, Gensini Score, Duke CAD Prognostic Index (DPI)], angiographic outcomes [change in minimum lumen diameter (∆MLD) and number of new lesions at follow-up], and key clinical outcomes (all-cause mortality, recurrent myocardial infarction and the need for coronary revascularization). We have for the first time confirmed an association of the allele with a higher Gensini score in a meta-analysis. In quantitative angiography Gensini score is derived by assigning a severity score to each coronary stenosis according to the degree of luminal narrowing and its geographic importance [28]. The score correlates positively with number of vessel segments involved. Thus it is intuitive that an association of 9p21-3 allele with triple vessel disease would translate into an association with the Gensini score in the same direction. However the lack of association with the DPI was surprising. A positive linear correlation between the Gensini and the DPI score is reported in the literature [9]. It is possible that the analysis of DPI was underpowered due to fewer studies reporting this association compared to those reporting the Gensini score.

We found no association between the 9p21-3 allele and angiographic outcomes. This was also unexpected as the process underlying de-novo atherogenesis would remain unchanged over the course of time. One likely explanation can be index event bias [36]. Conceivably, the risk factors distribution among patients with high genetic risk may have shifted after diagnosis and subsequent lifestyle modification and initiation of therapy.

We found no association between genotypic risk and all-cause mortality among CAD patients. This negative finding is supportive of existing evidence published by Ganna et al. [37] which showed that increased risk of all-cause mortality was associated with polygenic risk factors dispersed across the genome. In a sample of over 16,000 participants, a genome wide risk score derived from 707 published SNPs was associated with a modest 10% increased hazard of death. In our study we tested for association between a single locus in a smaller sample which could have further lowered the likelihood of finding an association.

Most GWAS showing locus-disease association, have shown positive results in conditions which are observed to be heritable. Given that there is no published study reporting heritability of the risk of re-infarction, the genetic risk of recurrent events among survivors of ACS remains less probable; an observation noted in our meta-analysis. Likewise in the case of TLR, which could result either from progression of atherosclerosis or recurrent acute ischemic events, we anticipated no association given the absence of increased risk of disease progression and re-infarction among 9p21-3 carriers.

Our study suffers some important limitations. First, we cannot rule out that our findings may be due to chance as multiple testing had been conducted. However, there is no consensus when this problem should be taken into account and which statistical method should be used in meta-analysis [38,39]. Second, the sample size in most of the outcomes reported in the studies was less than the OIS. Thus, we may not have the power to detect weaker associations. At last, our analyses restricted to association studies as no linkage analyses have identified this allele to be associated with CAD.

## Conclusion

Patients of CAD who carry the high risk genotype of the 9p21-3 allele may be more likely to have multi-vessel CAD. However the effect of this allele on CAD progression and disease specific clinical outcomes are not observed possibly due to diminishing genetic risk following dietary modification and therapy.

## Abbreviations

CAD: Coronary artery disease; GWAS: Genome wide association studies; MI: Myocardial infarction; PRISMA: The preferred reporting items for systematic reviews and meta-analyses statement; DPI: Duke CAD prognostic index; ∆ MLD: Change in minimum lumen diameter; ECG: Electrocardiography; LR: Homozygous low risk; IR: Heterozygous intermediate risk; HR: Homozygous high risk; WMD: Weighted mean difference; RR: Relative risk.

## Competing interests

The authors declare that they have no competing interests.

## Authors’ contributions

MM and ZW contributed equally to this study. MM carried out study design, study screening, data extraction, and drafted the manuscript. ZW carried out study design, quality appraisal, data analysis, and drafted the manuscript. FA participated in quality appraisal and critically revised the manuscript. MS conducted data extraction, drafted and critically revised the manuscript. PE designed the search strategy and revised the manuscript. IK carried out study design, advised on all methodological issues, drafted and critically revised the manuscript. MHM participated in study design, advised on all methodological issues, drafted and critically revised the manuscript. All authors approved the final version of this manuscript and agreed to be accountable for all aspects of the work

## Pre-publication history

The pre-publication history for this paper can be accessed here:

http://www.biomedcentral.com/1471-2350/15/66/prepub

## Supplementary Material

Additional file 1 Search Strategy. Click here for file
