# Supplementary material for: The association of 9p21-3 locus with coronary atherosclerosis: a systematic review and meta-analysis
Source: BMC Med Genet. 2014 Jun 6;15:66. doi: 10.1186/1471-2350-15-66 (PMC4074865; doi:10.1186/1471-2350-15-66)
Supplement: Additional file 1 — Search Strategy. [file 1471-2350-15-66-S1.docx]

**Additional file 1: Search Strategy**

PubMED (coronary disease OR coronary angiography OR angioplasty, balloon, coronary) AND 9p21* (English only) - 115

Ovid MEDLINE(R) 1948 to October Week 4 2011 # Searches Results Search Type

1. exp coronary disease

2. exp cardiac output, low/ or exp myocardial ischemia/ or exp ventricular dysfunction

3. 1 or 2

4. Chromosomes, Human, Pair 9

5. 3 and 4

6. 3 and 9p21*.mp. [mp=protocol supplementary concept, rare disease supplementary concept, title, original title, abstract, name of substance word, subject heading word, unique identifier]

7. 5 or 6

8. limit 7 to (english language and humans)

9. (gwa* or (genome adj wide) or cohort* or (case adj control*)).mp. [mp=protocol supplementary concept, rare disease supplementary concept, title, original title, abstract, name of substance word, subject heading word, unique identifier]

10. 8 and 9

11. 8 and eh.fs.

12. limit 8 to (comparative study or controlled clinical trial or meta analysis or multicenter study) 13. genome-wide association study/ or exp epidemiologic research design/ or exp genetic association studies

14. 8 and 13

15. 10 or 11 or 12 or 14

Embase 1988 to 2011 Week 44 # Searches Results Search Type

1. chromosome 9p

2. exp coronary artery disease

3. 1 and 2

4. 2 and 9p21*.mp. [mp=title, abstract, subject headings, heading word, drug trade name, original title, device manufacturer, drug manufacturer, device trade name, keyword]

5. 3 or 4

6. limit 5 to (human and english language)

7. (regist* or gwa* or (genome adj wide*)).mp. [mp=title, abstract, subject headings, heading word, drug trade name, original title, device manufacturer, drug manufacturer, device trade name, keyword]

8. 6 and 7

9. 6 and (population* or ethnic* or prevalence or cohort*).mp. [mp=title, abstract, subject headings, heading word, drug trade name, original title, device manufacturer, drug manufacturer, device trade name, keyword]

10. exp controlled study

11. 6 and 10

12. 8 or 9 or 11

Web of Science

Topic=((chr9p21* OR 9p21*) OR (9p SAME (allele* OR SNP*))) AND Topic=((myocardial OR coronary OR ventric* OR MI OR CAD OR CHD OR heart OR cardiovasc*) AND (gwa* OR "genome wide" OR population* OR cohort* OR ethnic* OR "case control*")) Refined by: Languages=( ENGLISH )

Scopus

(TITLE-ABS-KEY(((chr9p21* OR 9p21*) OR (9p AND (allele* OR snp*))) AND ((myocardial OR coronary OR ventric* OR mi OR cad OR chd OR heart OR cardiovasc*) AND (gwa* OR "genome wide" OR population* OR cohort* OR ethnic* OR "case control*"))) AND LANGUAGE(english)) AND PUBYEAR > 1999 AND NOT (PMID(1* OR 2* OR 3* OR 4* OR 5* OR 6* OR 7* OR 8* OR 9*))
